# Inheritance and Biological Characterization of an Orange-nymph Mutant in *Orius laevigatus* (Hemiptera: Anthocoridae)

**DOI:** 10.3390/insects13110996

**Published:** 2022-10-30

**Authors:** Amador Rodríguez-Gómez, Alberto Donate, Isabel Sánchez-Martínez, Virginia Balanza, Ana Belén Abelaira, María del Carmen Reche, Pablo Bielza

**Affiliations:** Departamento de Ingeniería Agronómica, Universidad Politécnica de Cartagena, 30203 Cartagena, Spain

**Keywords:** mutation, biological control agent, *Orius laevigatus*, body color, recessive allele

## Abstract

**Simple Summary:**

*Orius laevigatus* is a widely used predator and is key to the success of biological control programs. The nymphs of this predator are yellowish in all their nymphal stages. A nymph found in a mixture of wild populations did not present a normal coloration, but orange. A laboratory strain carrying this body color mutation *ambar* was established. The mutation (*ambar*) was controlled by a single autosomal recessive allele. Biological characteristics of the orange strain were compared with a normal population. The orange strain showed inferior performance in some traits, such as immature survival and development rate. Practical use of the orange mutation as a visible marker for biological and ecological studies of this important biological control agent is discussed.

**Abstract:**

A mutation showing a distinct orange color in the nymph stages was found in *Orius laevigatus* (Fieber) (Hemiptera: Anthocoridae), a key biological control agent in protected crops, used to control small pests, especially thrips. A laboratory strain carrying this body color mutation *ambar* was established. Genetic analysis determined that the mutation (*ambar*) was controlled by a single autosomal recessive allele. Some biological and ecological characteristics of this orange strain were compared to a normal population. Longevity, fecundity and fertility were similar in both populations, but immature survival, development rate, body size, starvation tolerance and predation capacity were inferior in the orange strain. The utility of the orange mutant as a visible marker for biological and ecological studies of this important biological control agent is discussed.

## 1. Introduction

*Orius laevigatus* (Fieber) (Hemiptera: Anthocoridae) is an important predator widely used in augmentative biological control programs, mainly to control thrips pests [1,2]. This biological control agent is mass produced to be released in different crops, particularly vegetable crops in greenhouses. This bug has been extensively studied in different laboratories to gain knowledge on their biology and ecology in order to improve their field performance and to optimize mass-rearing in biofactories [3,4,5,6,7,8,9,10,11,12,13,14,15]. Despite being reared by numerous biocontrol companies and researchers for many years, as far as we know, no mutations have been reported for this insect. A red-eye mutation has been described in adults of other *Orius* species, *O. sauteri* and *O. strigicollis* [16]. Mutations affecting external features such as body or eye color can be very useful as visible markers for multiple scientific studies [16,17,18,19]. Individuals carrying a visible mutation can be used to estimate the dispersal ability and the movement of this predator within and among plants, fields and crops. In addition, a visible marker can serve to compare establishment and efficiency of different strains released in a crop. A distinct body color can also serve for studies on mating and sexual competition. Furthermore, visible mutations can offer opportunities for analysis of insect genes and population genetics. Overall, strains of a biological control agent with genetically defined visible markers can be very useful to technical and scientific progress of biological control.

**Figure 1:**
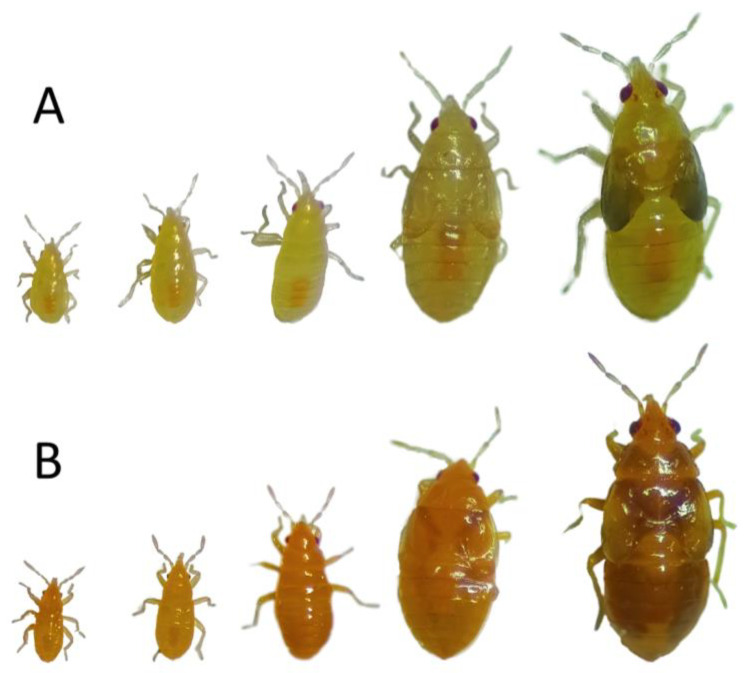
First to fifth instar nymphs of the normal (**A**) and mutant orange (**B**) populations of *Orius laevigatus*.

In 2021, one orange color nymph was found in a mixture of wild populations established in the Biocontrol Selection Lab (Universidad Politécnica de Cartagena, Cartagena, Spain) in the frame of a selective breeding program to select strains of *O. laevigatus* with enhanced characteristics [4], such as insecticide resistance [10,11,12,13], better fitness feeding on suboptimal food and larger body size [7,8,9]. Normal body color in *O. laevigatus* nymphs is yellowish, but this nymph exhibited a distinct orange body color (Figure 1). This nymph was used to establish a laboratory strain carrying this body color mutation *ambar*.

The present study aimed at studying the biological characteristics of this strain (body size, development, fecundity, starvation tolerance and predation) compared with a normal strain and to determine the inheritance of the *ambar* mutation.

## 2. Materials and Methods

### 2.1. Establishment of Orange Color Population

In 2021, we found a nymph showing a distinct orange body color in a mixture of wild populations established in the Biocontrol Selection Lab (Universidad Politécnica de Cartagena, Cartagena, Spain). In order to establish an orange mutant population (hereafter orange), we waited until the nymph reached adulthood and it turned out to be a female. The next step was to mate the orange virgin female with a normal male of *O. laevigatus* from a commercial population (hereafter normal) supplied by Biobest (Westerlo, Belgium). The offspring born from them were all normal nymphs. These normal F_1_ orius progeny were interbreed each other when they reached adulthood and produced both normal and orange nymphs. The orange nymphs (females and males) were selected in the third instar to establish a pure orange population.

### 2.2. Insect Rearing

Both the normal and orange populations were kept inside 1 L plastic containers with filter paper on the lid to allow ventilation. Inside each container, buckwheat husk was introduced to avoid cannibalism between individuals, *Ephestia kuehniella* eggs (hereafter *Ephestia*) ad libitum as food and pieces of green bean pods (*Phaseolus vulgaris* L.) were added as a source of moisture and oviposition site, previously treated with bleach and washed with water to remove any type of residue. Twice a week fresh food was added, and the bean was changed. The changed bean was separated and placed in different containers so that the nymphs did not mix with the adults in order to avoid cannibalism. Both populations (orange and normal) were maintained in the laboratory under the same conditions of 26 ± 1 °C, 65 ± 5% RH and 16:8 light—dark photoperiod.

### 2.3. Cross Experiments

A series of crosses were carried out to study the mode of inheritance of the mutation (*ambar*) involved in the orange coloration of *Orius* in their nymphal stages. Fifth instar nymphs were separated and individualized four days until they reached adulthood. To obtain the F_1_, virgin orange females and males were mated with virgin females and males from the normal population. A total of 30 crosses were made, 15 pairs of orange females and normal males and another 15 pairings of opposite sexes. The number of eggs was counted and separated by groups and female number (1 to 15). None of the nymphs born from these first crosses presented orange coloration. Once the nymphs hatched from these eggs reached the fifth instar, they were individualized and after four days, the backcrosses were carried out to each of the parental strains (orange and normal). In each cross, around 10–15 virgin females and the same number of virgin males were placed into 30 mL cups with *Ephestia* eggs as food and a 3.5 cm piece of green bean pod for oviposition. Twice a week, the bean pods were exchanged for new ones, and the eggs laid were counted. Stored individually in separate containers, the body color was recorded at the third instar nymph stage and then proceed to determine the orange and normal nymphs hatched from each cross. The rest of the females and males that were left over from the F_1_, after making the backcrosses, were interbred to produce F_2_. These cross experiments were conducted under the same conditions as the rearing, 26 ± 1 °C, 65 ± 5% RH and 16:8 light—dark photoperiod.

### 2.4. Fecundity, Fertility and Longevity Parameters

These experiments were conducted in order to compare egg production, hatching rate and longevity between the orange and the normal population. Fifth-instar nymphs were reared for each strain obtaining adult males and females after four days and during those days mating and pre-oviposition period took place. Exactly 40 females of each strain were sexed and isolated in a 30 mL plastic cup with ventilated lids, which contained *Ephestia* eggs ad libitum as food and a 3.5 cm piece of green bean pod sealed with paraffin at both ends for hydration and oviposition. Twice a week, eggs were counted, examining each piece of green bean pod using a stereoscopic microscope and switching the piece to a clean one and adding fresh food. This process was repeated until the female died; in this way, its longevity was measured. To obtain fertility data, the eggs of the first two fecundity counts were kept individually in laboratory conditions. Hatched eggs were counted after four days. The hatching percentage was calculated dividing the number of eggs that hatched by the total number of eggs. These experiments were conducted under 26 ± 1 °C, 65 ± 5% RH and 16:8 light—dark photoperiod.

### 2.5. Immature Development

For this type of experiment, to collect fresh eggs from both populations, small pieces of bean pods (maximum 3 cm) were introduced into the containers with adults of each population (normal and orange). The bean pod sections were changed every 24 h for a week. In order to obtain the high number of fresh eggs necessary for the experiment, several extractions of eggs were carried out and the bean pod sections removed each day were put at 6 °C in the refrigerator to avoid the development of the eggs. At the end of the week (four changes of bean pods), all the sections of bean pods with eggs were put at 26 ± 1 °C, 65 ± 5% RH and 16:8 light—dark photoperiod. The eggs of each piece of bean were counted using a stereoscopic microscope and every 90–110 eggs were placed in 200 mL cardboard cups with a piece of bean of approximately 5 cm and sealed on both sides, buckwheat husk and *Ephestia* eggs ad libitum, thus forming a replication. Each cup containing 90–100 eggs was considered a replication, totaling five replications for each population (a total of around 500 eggs per population). The change of bean pods and the supply of fresh food was made three times a week, always observing the nymphal stage of the individuals born in each cup. After 10 days, the cups were observed every 24 h to remove newly emerged adults (<24 h), following this procedure until there were no nymphs left. All adults were frozen. The width of the pronotum was measured in all individuals with a stereoscopic microscope at 50×, differentiating between males and females.

### 2.6. Starvation Tolerance

With the aim of assessing the quality of the adults from the orange and the normal populations, the tolerance to the lack of food (starvation) was tested. For this experiment, fifth instar nymphs of both populations were selected and left for four days to reach adulthood. The adults were sexed, and males were separated from females, leaving each group by sex and population in its corresponding 200 mL cardboard cup. The total number of individuals introduced into each cup was between 65 and 100. Each cup contained a piece of bean pod of about 5 cm sealed on both sides with paraffin and buckwheat hull. No food was introduced at any time during the experiment. Mortality was assessed every 24 h until there was no one alive. This experiment was conducted under 26 ± 1 °C, 65 ± 5% RH and 16:8 light—dark photoperiod.

### 2.7. Predation Capacity

The predation capacity between the orange strain and the normal population was compared. As prey, 30 adults of *Frankliniella occidentalis* (Pergande) (Thysanoptera: Thripidae) from a laboratory colony were used for each replicate. Pepper leaves were cut into elongated sections of about 30 × 5 mm and filled into 5 mL vials. The densities of 30 thrips per vial were evaluated, including one female of *O. laevigatus* with a previous fast of 24-h. The number of replications per population of *O. laevigatus* was twenty. A negative control of ten replicates and ten thrips per replicate was also evaluated to test survival in the absence of a predator. After 24 h, the predators were removed from the vials and the predated prey were counted. The dead individuals were also counted in the vials without predators (negative controls). This experiment was conducted under 26 ± 1 °C, 65 ± 5% RH and 16:8 light—dark photoperiod.

### 2.8. Data Analysis

A chi-square analysis was used to compare the proportions of body color (orange:normal) against the expected ratios of 1:3 for F_2_ crosses and 1:1 for backcrosses. Differences in biological and ecological characteristics between both populations were analyzed using a one-way analysis of variance. Assumptions of normality and homogeneity of variances were checked prior to the analysis. When significant differences between populations were observed, the means were separated using Tukey’s HSD test.

## 3. Results

### 3.1. Establishment of the Orange Population

**Figure 2:**
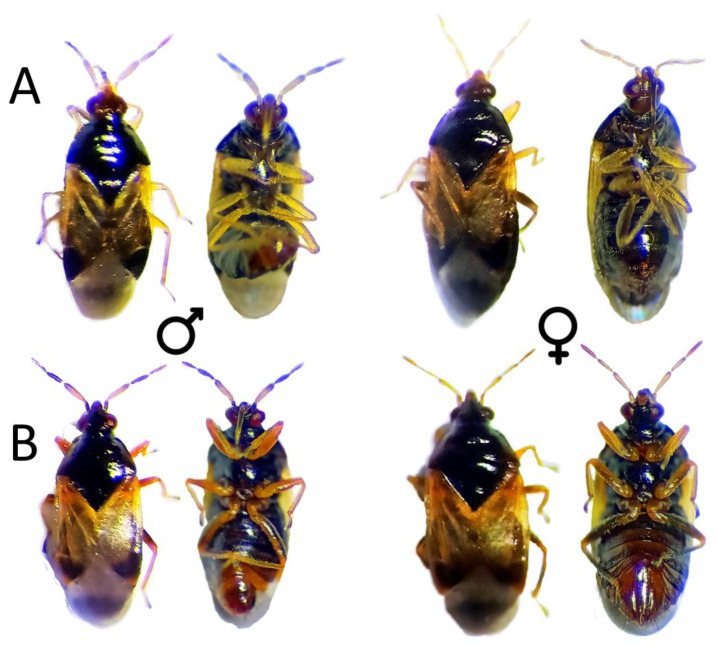
Recently emerged normal (**A**) and orange mutant (**B**) male and female adults of *Orius laevigatus*.

Through a selection of nymphs descending from the F_2_ of an original orange nymph, a population carrying the *ambar* mutation was established. All the individuals of the population could be easily discriminated from a normal population in each nymphal stage (from the first stage to the fifth) (Figure 1), presenting an orange coloration very different from the yellowish color typical in a normal population (Figure 1). Upon adult emergence, the orange coloration can be seen during the first hours as an adult. As the hours go by, the head, thorax and abdomen turn dark, similar to that of a normal *O. laevigatus* (Figure 2). The adults (males and females) with several days did not present appreciable differences between populations (orange and normal) with the naked eye, but observing through a stereoscopic microscope, the legs present an orange coloration that can be differentiated in those individuals coming from the orange population (Figure 2).

### 3.2. Cross Experiments

The results between the orange and normal crosses presented an offspring with wild-type color (F_1_), except for those crosses when both the male and the female were orange (Table 1). Inbreeding between F_1_ individuals (ONxON and NOxNO) produced F_2_ offspring, presenting the expected ratio 1:3 (Table 1). The same happened in the case of backcrosses using parents, the offspring fitted with the expected ratio 1:1 (Table 1).

### 3.3. Fecundity, Fertility and Longevity Parameters

The results for fecundity, fertility (percentage of eggs hatched) and longevity of females of the orange and normal populations are shown in Table 2. Both populations presented similar values for fecundity (F = 0.05, df= 1/68, *p* > 0.05), fertility (F = 0.42, df = 1/60, *p* > 0.05), and longevity (F = 0.01, df= 1/75, *p* > 0.05).

### 3.4. Immature Development

The results of survival and duration of immature development from egg to adult are summarized in Table 2. The percentage of survival was much lower in the orange than in the normal population (F = 98.3, df = 1/18, *p* < 0.001). Similarly, the development time in the orange population was longer, thus, taking longer from hatching to adult than in the normal population (F = 170.09, df = 1/18, *p* < 0.001). The body size (width of the pronotum) of orange adult females and males resulted significantly inferior to those of the normal population (F = 90.1, df = 1/574, *p* < 0.001) (Table 3).

### 3.5. Predation capacity

Prey consumption differed significantly between the orange and normal populations when preying on adult thrips (F = 21.57, df = 1/38, *p* < 0.001). The orange population presented lower consumption of adults than the normal populations (19.2 vs. 23.3 thrips adults) (Table 4).

### 3.6. Starvation Experiments

Adult females of the normal population lived longer than those of the orange population when deprived of food (F = 5.68, df = 1/165, *p* < 0.05) (Table 4).

## 4. Discussion

Experimental crosses indicated that the *ambar* mutant of *O. laevigatus* was controlled by a single autosomal recessive allele. Body color mutations have been studied in other heteropteran bugs, particularly in *Pyrrhocoris apterus* L. (Hemiptera: Pyrrhocoridae) [20]. Several mutations affecting body color (white, yellow and melanotic) of *P. apterus* were inherited as single autosomal recessive genes [17]. An orange body mutant was reported in the cotton stainer *Dysdercus koenigii* F. (Hemiptera: Pyrrhocoridae) as governed also by a single somatic recessive gene [21]. No mutations affecting body color have been reported in any *Orius* species, but a red-eye mutation in *O. sauteri* and *O. strigicollis* was also found to be autosomal, single-locus and recessive [16].

Life-time fecundity, egg hatching and life span were similar in both the orange and normal populations, and within the range reported in other studies [5,7,8,14,15,22,23,24,25]. However, the orange population showed a significantly reduced survival from egg to adult (37%) and delayed development (14.5 days) compared to those of the normal population (89% and 12.7 days, respectively).

On the other hand, predation rate, starvation tolerance and body size are considered traits especially critical for effective biological control [26]. The orange population underperformed the normal population in all these three key traits. Both starvation tolerance and predation rate are related to body size, as it has a profound effect on most biological and ecological traits [27,28,29]. Large individuals are expected to have more body reserves, resulting in increased starvation tolerance. Moreover, body size is particularly prominent for a predator, since it is strongly related to the range of prey is capable of attacking [29]. In fact, a strain of *O. laevigatus* artificially selected for larger body size exhibited a superior predation capacity on thrips than commercial and wild populations [9]. Therefore, the smaller body size of the orange population is likely related to the inferior starvation tolerance and predation capacity.

The poor performance of the orange population in some traits is not necessarily associated with the *ambar* mutation, since it might be attributed to natural differences among populations of the same species, as already reported in *O. laevigatus* [7,8,9]. Moreover, the orange population originated from a single orange female, and it is very likely to suffer from inbreeding depression, unmasking deleterious recessive alleles [30,31]. Therefore, before further use of this mutation in biological and ecological studies of *O. laevigatus*, it will be needed to repeatedly backcross the orange population with other populations to find a population carrying the *ambar* mutation but exhibiting similar fitness to standard populations.

The orange body mutation in *O. laevigatus* is a very useful visible marker for biological and ecological studies of this important biological control agent. Since the strain orange carrying the *ambar* mutation seems to show worse performance in some biological traits (immature development, body size and predation capacity), the mutation should be introgressed in well-performing strains. Strains carrying and not the *ambar* mutation can be employed in studies to compare establishment and biocontrol services of different strains of this predator. Indeed, these strains can be utilized to monitor field performance of genetically improved strains of *O. laevigatus*, such as those selected for insecticide resistance [10,11,12,13], better fitness feeding on suboptimal food and larger body size [7,8,9]. Similarly, this orange strain can serve in research on dispersal and distribution within and among plants, crops and fields of *O. laevigatus,* avoiding the risk of mistaking the individuals released with naturally occurring populations. Knowledge of movement and population dynamics of this predator would be very beneficial for the implementation of integrated pest management programs and, in particular, augmentative and conservative biological control protocols. In laboratory works, the *ambar* mutation can be utilized in studies on paternity and mating, comparing sexual competition of different strains. Moreover, a visible mutation such as *ambar* can be a valuable tool for research on genomics and population genetics of this important natural enemy. In conclusion, the *ambar* mutation in *O. laevigatus* can offer interesting opportunities to technical and scientific progress of the use of this biological control agent.

Finding out the actual mutation with the help of the first genome assembly for *O. laevigatus* [32] and the mechanism involved would likely be the next best steps to fully understand this mutation.

## Figures and Tables

**Table 1:** Nymphs color in offspring of crosses and backcrosses between mutant (orange) and wild (normal) *Orius laevigatus* populations.

Cross (Female × Male)	Orange (O)	Normal (N)	Expected Ratio (O:N)	Observed Ratio (O:N)	χ^2^	*p*
O × N (15)	0	67	0:1			
N × O (15)	0	237	0:1			
O × O (15)	77	0	1:0			
ON (61) × ON (37)	294	789	1:3	1:2.68	2.549	0.110
NO (56) × NO (45)	116	418	1:3	1:3.60	2.886	0.086
ON × OO (14)	176	157	1:1	1:0.89	0.973	0.324
OO × ON (10)	98	79	1:1	1:0.81	1.813	0.176
NO × OO (15)	216	204	1:1	1:0.94	0.288	0.591
OO × NO (15)	44	28	1:1	1:0.64	3.125	0.077

The number in brackets is the number of couples used in each cross. The offspring of each cross were recorded in third instar nymph stages. NO: offspring of wild (normal) females and mutant (orange) males; ON: offspring of mutant (orange) females and wild (normal) males.OO: offspring of mutant (orange) females and mutant (orange) males.

**Table 2:** Fecundity, fertility, longevity, immature survival and development time from egg to adult in the orange (mutant) and normal (wild) populations of *Orius laevigatus*.

Type	Fecundity (Eggs/Female)	Fertility (% Eggs Hatched)	Longevity (Days)	Immature Survival (%)	Development Time (Days)
Orange	99.7 ± 10.0 a	92.1 ± 1.5 a	24.1 ± 2.7 a	36.8 ± 2.9 a	14.5 ± 0.11 b
Normal	96.4 ± 10.9 a	93.4 ± 1.4 a	23.7 ± 2.8 a	89.1 ± 4.4 b	12.7 ± 0.07 a

Means ± SE within a column followed by the same letter are not significantly different (*p* > 0.05; Tukey test).

**Table 3:** Mean body size (width of pronotum) of orange and normal adult males and females.

Sex	Population	Pronotum Size (mm)
Female	Orange	0.78 ± 0.002 a
	Normal	0.80 ± 0.001 b
Male	Orange	0.74 ± 0.003 a
	Normal	0.76 ±0.001 b

Means ± SE within each sex followed by the same letter are not significantly different (*p* > 0.05; Tukey test).

**Table 4:** Prey consumption by adult females of the orange (mutant) and normal (wild) populations of *Orius laevigatus*.

Population	Prey Offered	# Prey Consumed	% Prey Consumed	Longevity under Starvation (Days)
Orange	30	19.2 ± 0.71 a	64.0	3.1 ± 0.22 a
Normal	30	23.3 ± 0.50 b	77.5	4.1 ± 0.39 b

Means ± SE within each column followed by the same letter are not significantly different (*p* > 0.05; Tukey test).

## Data Availability

The datasets generated and/or analysed during the current study are available from the corresponding author on reasonable request.
